# Chemometrics Combined with Multi-Source Spectroscopy for Fruit Germplasm Quality Evaluation: A Case Study on Quince (*Cydonia oblonga*)

**DOI:** 10.3390/foods15142558

**Published:** 2026-07-21

**Authors:** Zhenzhen Ding, Tingting Su, Xia Zhang, Li Wang, Xueqing Wang, Chao Li, Yutao Wang

**Affiliations:** 1 College of Life and Geography Sciences, Xinjiang Key Laboratory of Biological Resources and Ecology of Pamir Plateau, Kashi University, Kashi 844000, Chinafelichao@scut.edu.cn (C.L.); 2 Graduate School of Medicine, Hokkaido University, Kita-9 Nishi-9, Kita-ku, Sapporo 060-8589, Japan; 3 School of Food Science and Engineering, Zhuhai Institute of Modern Industrial Innovation, South China University of Technology, Guangzhou 510640, China

**Keywords:** *Cydonia oblonga* Mill., electronic nose, HS-SPME-GC-MS, chemometrics, quality profiling, volatile organic compounds

## Abstract

Quince (*Cydonia oblonga* Mill.) is an important fruit crop, yet a systematic quality evaluation framework is lacking. This study comprehensively characterized multiple germplasms from distinct production areas. An integrated strategy combining physicochemical analysis, FT-MIR (Fourier transform mid-infrared spectroscopy), electronic nose (E-nose), headspace solid-phase microextraction–gas chromatography–mass spectrometry (HS-SPME-GC-MS), and chemometrics was employed. Significant variations were observed among accessions: certain varieties were observed to exhibit the highest pectin (1.86%) and total phenolic content (143.40 mg/100 g), while others showed superior firmness and titratable acidity. β-Damascenone in one accession was found to have an exceptionally high odor activity value (OAV) of 265.05. Multivariate analysis, including PLS-DA and OPLS-DA, effectively discriminated among quince accessions, with PLS-DA achieving 100% classification accuracy, and tentatively identified 18 key markers (VIP > 1) for accession discrimination. Loading scatter plot analysis further validated the contribution of these markers to the separation between accessions. However, due to the limited sample size (*n* = 18) and partial confounding between cultivar and origin, these findings should be considered exploratory and require validation in larger independent studies. This work provides preliminary insights into the diversity and geographical patterns of quality and flavor traits in quince germplasm, offering a preliminary foundation for germplasm evaluation and targeted utilization.

## 1. Introduction

Quince (*Cydonia oblonga* Mill.), a deciduous tree within the Rosaceae family, is a characteristic fruit crop in Xinjiang, China. The fruit is rich in bioactive compounds such as polyphenols, carotenoids, and pectin, which confer various health-promoting properties including antioxidant, anti-inflammatory, and hypoglycemic activities, highlighting its potential in the functional food and nutraceutical sectors [1,2,3]. According to the Food and Agriculture Organization (FAO), global quince production reached approximately 700,000 tons in 2020, with Turkey and China jointly accounting for 43%. Notably, Xinjiang quince, adapted to the unique arid climate, has developed rich germplasm resources, granting it importance within the global quince repository.

The nutritional quality and chemical composition of quince are known to be influenced by both genotype (cultivar) and geographical origin (growth conditions), leading to substantial variations [4,5]. Different cultivars exhibit marked differences in fruit size, weight, and the composition of functional and flavor components [6]. For instance, oblong-shaped quinces can contain up to twice the phenolic content of apple-shaped varieties [7]. Chlorogenic acid levels vary significantly among cultivars [8], and GC-MS analyses confirm distinct cultivar-specific volatile profiles [9]. The influence of geographical origin is equally pronounced, with studies showing that phenolic profiles in quince juice vary significantly across production areas, and local soil and climatic conditions can alter both phenolic composition and physicochemical properties [10,11].

Despite these recognized influences, critical scientific gaps remain in the context of Xinjiang quince. Firstly, most existing research focuses on isolated components, leaving the potential associations between multiple bioactive compounds and their collective contribution to overall antioxidant efficacy poorly understood [6,12,13]. Secondly, systematic investigations linking geographically characteristic flavor profiles with underlying chemical composition are scarce, which hinders the scientific basis for developing and protecting geographical indication products. Furthermore, conventional univariate analytical methods are inadequate for the comprehensive, multidimensional quality assessment required to address these complex questions.

In contrast, the integration of multi-source spectroscopy (e.g., FT-MIR) with advanced chemometrics offers a potential approach for the rapid, non-destructive, and simultaneous analysis of multiple quality indicators, enabling the identification of key markers for origin discrimination and quality prediction.

Therefore, this study was designed to systematically investigate six representative quince germplasms from four major producing areas in Xinjiang. An integrated approach combining physicochemical analysis, FT-MIR, electronic nose (E-nose), headspace solid-phase microextraction–gas chromatography–mass spectrometry (HS-SPME-GC-MS), and multivariate statistical analysis was employed. The objectives were to (1) investigate the geographical variation patterns in quality (e.g., pectin, phenolics, antioxidant capacity) and flavor traits, and identify the potential key chemical drivers; (2) explore the component basis and potential interactions underlying antioxidant activity; and (3) screen distinctive germplasms with high potential for targeted, value-added development. This work provides a preliminary theoretical foundation for precise germplasm identification, quality improvement, and high-value utilization of quince. It is important to note that due to the limited sample size (*n* = 6 accessions, 18 samples) and partial confounding between cultivar and geographical origin, the findings presented in this study are primarily exploratory and require validation in future research with larger, independent sample sets.

## 2. Materials and Methods

### 2.1. Materials and Reagents

#### 2.1.1. Plant Materials and Sampling

Six distinct quince accessions were collected at commercial maturity (September–October 2025) from four major producing regions in Xinjiang, China. These regions are located in the southwestern and southern parts of Xinjiang (the relative geographical locations are shown in Figure S1 of the Supplementary Materials). For each accession, 10 uniform, defect-free fruits were harvested from multiple trees (or different positions within the same orchard) to capture the natural intra-accession variability. Details are as follows: from Hotan City (37°11′ N, 79°50′ E), apple-shaped quince (LXH, *C. oblonga* var. maliformis); from Zepu County (38°15′ N, 77°20′ E), pear-shaped (YQZ, *C. oblonga* var. pyriformis), apple-shaped (LXZ, *C. oblonga* var. maliformis), and large-fruited quince (BQZ, *C. oblonga* var. lusitanica); from Kashi City (39°28′ N, 75°59′ E), pyramidalis quince (LQK, *C. oblonga* var. pyramidalis); and from Shule County (39°25′ N, 76°06′ E), apple-shaped quince (LXS, *C. oblonga* var. maliformis). It is important to acknowledge a limitation of this sampling design: different varieties were collected from the same site (e.g., Zepu County contributed three varieties), while some origins were represented by only a single variety (e.g., Hotan, Kashi, and Shule). This partial confounding between cultivar and geographical origin makes it difficult to fully separate the effects of genotype from those of geographical origin. Therefore, a dedicated “Limitations of the study”, Section 4.5, has been added to the Discussion to explicitly address this concern. Uniform, defect-free fruits were selected, transported to the laboratory, and stored at 4 °C for 48 h prior to analysis to simulate standard postharvest handling.

#### 2.1.2. Instruments and Reagents

The antioxidant activity assays for superoxide anion (O_2_^−^·), ferric reducing antioxidant power (FRAP), and 2,2-diphenyl-1-picrylhydrazyl (DPPH) radical scavenging capacity were performed using commercial kits (Solarbio, Beijing, China). 2-Octanol (≥99% purity), which was used as an internal standard for volatile analysis, was obtained from Sigma-Aldrich (St. Louis, MO, USA). All other chemicals and solvents used were of analytical grade and purchased from Sinopharm Chemical Reagent Co., Ltd. (Shanghai, China).

### 2.2. Experimental Methods

#### 2.2.1. Analysis of Appearance and Physicochemical Quality

Fruit weight was determined using an electronic analytical balance (precision: 0.01 g). For each accession, 8–10 fruits were randomly selected, and the mean value was calculated. Color parameters (L^*^, a^*^, b^*^) were measured using a colorimeter at three equatorial points per fruit, with five biological replicates per accession [14]. Firmness and chewiness were evaluated via texture profile analysis using a texture analyzer equipped with a TA/2N cylindrical probe. The test conditions were set as follows: pre-test speed 2.0 mm/s, test speed 1.0 mm/s, post-test speed 1.0 mm/s, deformation 30%, trigger force 5 g, and a 5 s interval between two compressions.

#### 2.2.2. Basic Chemical Composition

Soluble solid content (SSC) was measured using a handheld refractometer. Titratable acidity was determined by titration with 0.1 mol/L NaOH and expressed as malic acid equivalent (conversion factor K = 0.067) [15]. Pectin content was quantified by the citric acid extraction-ethanol precipitation gravimetric method. Crude fiber content was assessed via the standard gravimetric method [16].

#### 2.2.3. Extraction and Determination of Bioactive Compounds

Total phenolic content was determined using the Folin–Ciocalteu method with gallic acid as the standard, and the absorbance was measured at 765 nm [17]. Prior to analysis, samples were extracted with a water:methanol mixture (2:8, *v*/*v*) [18]. Total flavonoid content was analyzed by the aluminum chloride colorimetric assay, measuring the absorbance at 510 nm [19]. Total carotenoid content was quantified after ultrasonic extraction with an acetone-petroleum ether mixture (1:2, *v*/*v*), followed by spectrophotometric measurement at 450 nm [20].

#### 2.2.4. Determination of Antioxidant Capacity and Related Enzyme Activities

The antioxidant capacities, including DPPH and ABTS radical scavenging activities, as well as ferric reducing antioxidant power (FRAP), were assessed using commercial assay kits. All procedures were performed strictly according to the manufacturers’ protocols, and absorbance was recorded using a microplate reader [21]. The activities of superoxide dismutase (SOD) and peroxidase (POD) were also determined using specific assay kits, with the results expressed in units per gram of sample (U/g) [22].

#### 2.2.5. Near-Infrared Spectroscopy Analysis

Fruit samples were transversely sliced into sections of approximately 2–3 mm thickness. FT-MIR spectra were acquired using a Fourier transform infrared spectrometer (Thermo Fisher Scientific, Waltham, MA, USA; Model Nicolet 5700) over the full wavenumber range of 400–4000 cm^−1^ with a resolution of 4 cm^−1^. To better visualize the characteristic absorption peaks (e.g., C=O at ~1650 cm^−1^ and C–O at ~1050 cm^−1^), the 800–2000 cm^−1^ region was selected for analysis and graphical presentation. Each sample was scanned three times, and the average spectrum was used for subsequent analysis to ensure reproducibility [23].

#### 2.2.6. Electronic Nose Analysis

The global volatile profile was assessed using an electronic nose (E-nose) system (Airsense Analytics GmbH, Schwerin, Germany; Model PEN 3). Precisely 10.00 g of homogenized pulp was sealed in a 20 mL headspace vial and equilibrated at 25 °C for 30 min. The measurement was conducted at a sampling flow rate of 400 mL/min over a period of 120 s. All analyses were performed in triplicate [24].

#### 2.2.7. HS-SPME-GC-MS Analysis and Odor Activity Value Calculation

Volatile compounds were identified and quantified using a HS-SPME-GC-MS system (Shimadzu Corporation, Kyoto, Japan; Model GCMS-QP2020NXNC). A pulp sample (10.00 g) was placed in a 20 mL headspace vial, mixed with 2 g of NaCl and 5 μL of 2-octanol (internal standard, 20 mg/L in methanol), and the vial was immediately sealed. The HS-SPME fiber (50 μm DVB/CAR/PDMS) was conditioned at 250 °C for 30 min before the first use and reconditioned at 250 °C for 5 min between each extraction. The sample vial was equilibrated at 40 °C for 15 min, followed by extraction for 35 min at 40 °C. The fiber was then desorbed in the GC injector port at 250 °C for 3 min in splitless mode.

Chromatographic separation was achieved using a DB-5 capillary column (60 m × 0.25 mm × 0.25 μm; Agilent Technologies, Santa Clara, CA, USA) with helium as the carrier gas at a constant flow rate of 1.0 mL/min. The oven temperature was programmed as follows: it was held at 40 °C for 2 min, then ramped to 230 °C at a rate of 3 °C/min, and finally held at 230 °C for 5 min. The mass spectrometry conditions were as follows: electron impact (EI) ionization source at 70 eV, ion source temperature of 230 °C, and a scan range of *m*/*z* 35–500.

The odor activity value (OAV) was calculated as the ratio of the concentration of a volatile compound (C) to its odor threshold in water (T) (OAV = C/T). Compounds with an OAV ≥ 1 were considered key aroma-active contributors [25].

### 2.3. Data Processing

All data are presented as mean ± standard deviation. Statistical analysis was performed as follows: (1) Univariate analysis: One-way analysis of variance (ANOVA) followed by Duncan’s multiple range test (*p* < 0.05) was conducted using SPSS (Version 26.0; IBM Corp., Armonk, NY, USA) to determine significant differences among groups. (2) Correlation analysis: Pearson correlation analysis (*p* < 0.05) was performed using OriginPro 2024 (OriginLab Corp., Northampton, MA, USA). (3) Data fusion strategy: To integrate multiple data sources (physicochemical properties, FT-MIR spectra, electronic nose responses, and volatile profiles), a mid-level data fusion approach was adopted. First, features were independently selected from each data block based on VIP > 1.0 and significance in univariate analysis. Second, all selected features were block-wise scaled (autoscaling per block) to avoid block dominance. Finally, the fused feature matrix was subjected to OPLS-DA. (4) Multivariate modeling: Two complementary multivariate modeling strategies were employed. First, PLS-DA was applied to evaluate the overall discriminative power among the four quince accessions, as this is the standard method for multi-class classification problems. Second, OPLS-DA was performed for pairwise comparisons between specific accessions (e.g., LQK vs. LXZ) to identify accession-specific marker compounds, which is the established application of OPLS-DA. All multivariate analyses were performed using SIMCA (Version 14.1; Sartorius Stedim Data Analytics AB, Umeå, Sweden). Given the small sample size (*n* = 18), to minimize the risk of overfitting, the model was validated using the following conservative strategies: Leave-one-out cross-validation (LOOCV) was performed to compute the Q^2^ value. Permutation testing (200 permutations) was conducted to verify model validity. The R^2^Y and Q^2^ values were reported and compared with those of single-block models to assess the benefit of data fusion. Variables with a VIP value > 1.0 in the fused model were considered key differential markers. It is important to note that due to the limited number of accessions (*n* = 6, 3 replicates each) and the partial confounding between cultivar and geographical origin, the model is primarily exploratory and should be validated in future studies with larger, independent sample sets. Prior to OPLS-DA, all variables were autoscaled (Unit Variance scaling) using the default settings in SIMCA (Version 14.1).

## 3. Results

### 3.1. Significant Chemodiversity in Morphological and Physicochemical Traits Reveals Cultivar and Origin Effects

The external morphology and basic physicochemical characteristics serve as primary indicators for evaluating commercial value and processing suitability. Systematic analysis revealed significant differences in these traits among cultivars and geographical origins (Table 1), indicating substantial environmental influences. The external morphology and basic physicochemical characteristics serve as primary indicators for evaluating commercial value and processing suitability. Systematic analysis revealed significant differences in these traits among cultivars and geographical origins (Table 1), indicating substantial environmental influences.

#### 3.1.1. Diversity in Fruit Morphology and Color Characteristics

Fruit shape, a genetically stable trait, exhibited significant variation among the tested accessions. The shape index ranged from 0.88 (BQZ) to 1.01 (LQK), indicating a morphological continuum from oblate (BQZ, LXS) to nearly round (LXH, LQK). Notably, LXZ was characterized by a significantly greater longitudinal diameter, resulting in a typical pyriform shape, whereas LQK exhibited a near-spherical morphology.

Fruit color parameters also showed significant diversity. Regarding lightness, LXZ displayed the highest L^*^ value, while LXS showed the lowest, indicating a stark contrast in peel brightness. In terms of chromaticity, YQZ and LXH possessed the highest a^*^ (redness) values. Concurrently, LXH also recorded the highest b^*^ (yellowness) value.

#### 3.1.2. Variations in Textural Properties and Flavor Components

Textural profiling revealed significant differences in fruit firmness and chewiness among the accessions, which are closely related to cellular structure. LQK and LXS exhibited the highest values for both hardness and chewiness, suggesting a more compact cell wall architecture. This structural characteristic is advantageous for postharvest handling and transport but may concurrently limit their fresh-eating palatability.

The contents and ratios of soluble solids and titratable acidity, which are fundamental to the sweet-sour taste balance, also varied markedly among the germplasms. LQK possessed the highest soluble solid content, whereas LXZ registered the highest titratable acidity. These variations directly resulted in a significant divergence in the sugar-to-acid ratio, thereby shaping distinct flavor profiles. Accessions with higher ratios presented a sweetness-dominant, mildly acidic flavor profile that is preferred for fresh consumption. In contrast, those with lower ratios exhibited a pronounced sourness, indicating a potential advantage for the development of processed products requiring high acidity, such as jams and fruit vinegars.

In summary, this study identified significant genetic differentiation in morphology, texture, and flavor composition among quince germplasms. These systematic variations, exemplified by the large size and high acidity of LXZ, the small size, high sweetness, and hardness of LQK, and the high firmness and tartness of LXS, establish a solid foundation of physicochemical traits for the precise selection and targeted utilization of germplasm resources according to specific processing objectives and consumer markets.

### 3.2. Variations in Bioactive Compounds and a Complex, Non-Linear Antioxidant System

The content of major bioactive compounds varied significantly (*p* < 0.05) among the quince cultivars (Table 2), indicating that genetic background is a key determinant in the accumulation of these functional components.

#### 3.2.1. Significant Differences in Bioactive Compound Contents

Among the compounds analyzed, total phenolic and flavonoid contents exhibited the most pronounced variation. The total phenolic content was highest in LXS, with its value approximately three times that of the cultivar with the lowest level. In contrast, LXZ accumulated the highest level of total flavonoids. Carotenoid content also differed notably among the accessions, with LXH showing the most prominent accumulation. In comparison, anthocyanin content showed relatively minor variation across the germplasms, which may be attributed to the generally low basal level and a more conserved biosynthetic pathway for anthocyanins in quince fruits.

These germplasm-specific distribution patterns of bioactive constituents not only reflect the differential potential for secondary metabolite biosynthesis among genetic resources, but also provide a direct chemical basis for the subsequent selection of germplasms targeting specific health-promoting effects (e.g., antioxidant, anti-inflammatory) and for the development of value-added products.

#### 3.2.2. Method-Dependent Variations in Antioxidant Capacity

The antioxidant capacity of quince fruits was evaluated using multiple in vitro chemical assays, and the results revealed that the measured activity was significantly method-dependent, while also exhibiting a distinct variation pattern among the different cultivars (Table 2). Specifically, LXH and LXZ demonstrated the highest scavenging activity against the ABTS^+^• radical, a method primarily sensitive to hydrophilic antioxidants. In contrast, LQK exhibited the most potent capacity to scavenge the DPPH• radical, a model commonly associated with lipophilic antioxidants. For the FRAP, which measures electron-donating capacity, LXZ showed the strongest reducing potential. Notably, in the assay for T-AOC, which integrates multiple reaction mechanisms, BQZ recorded the highest value. Collectively, these findings indicate that the differential efficacy profiles of various quince cultivars against distinct oxidative stress models are likely attributable to their specific compositions of antioxidant compounds, such as hydrophilic phenolics, lipophilic constituents, or particular reducing agents. The superior performance of BQZ in the comprehensive T-AOC assay suggests it may possess a broader spectrum or a more synergistic combination of antioxidative components.

#### 3.2.3. Distinct Patterns in Antioxidant Enzyme Activities

In addition to endogenous non-enzymatic antioxidants, the fruit’s antioxidant system comprises a suite of key antioxidant enzymes. This study revealed that the activities of three major antioxidant enzymes—PPO, POD, and SOD—varied significantly among the quince accessions and exhibited distinct germplasm-specific patterns (Table 2). Among them, PPO activity peaked in LXH. Notably, POD activity showed the greatest variation, with the level in YQZ being exceptionally high, nearly an order of magnitude greater than that in other accessions. Furthermore, YQZ also exhibited the highest SOD activity among all cultivars.

The patterns of enzyme activity did not entirely align with the distribution profiles of the non-enzymatic antioxidants described earlier. For instance, while YQZ possessed the highest POD and SOD activities, its total phenolic content was relatively low. Conversely, LXS, which had the highest total phenolic content, did not show a correspondingly high level in major enzyme activities. This decoupling between the non-enzymatic and enzymatic antioxidant systems suggests that different quince cultivars may have evolved distinct strategies for oxidative stress response and metabolic regulation. The exceptionally high POD activity in YQZ is particularly noteworthy, potentially indicating unique physiological traits in its fruit tissues for coping with peroxide stress or in specific secondary metabolic pathways.

#### 3.2.4. Correlation Analysis Between Bioactive Compounds and Antioxidant Capacity

**Figure 1:**
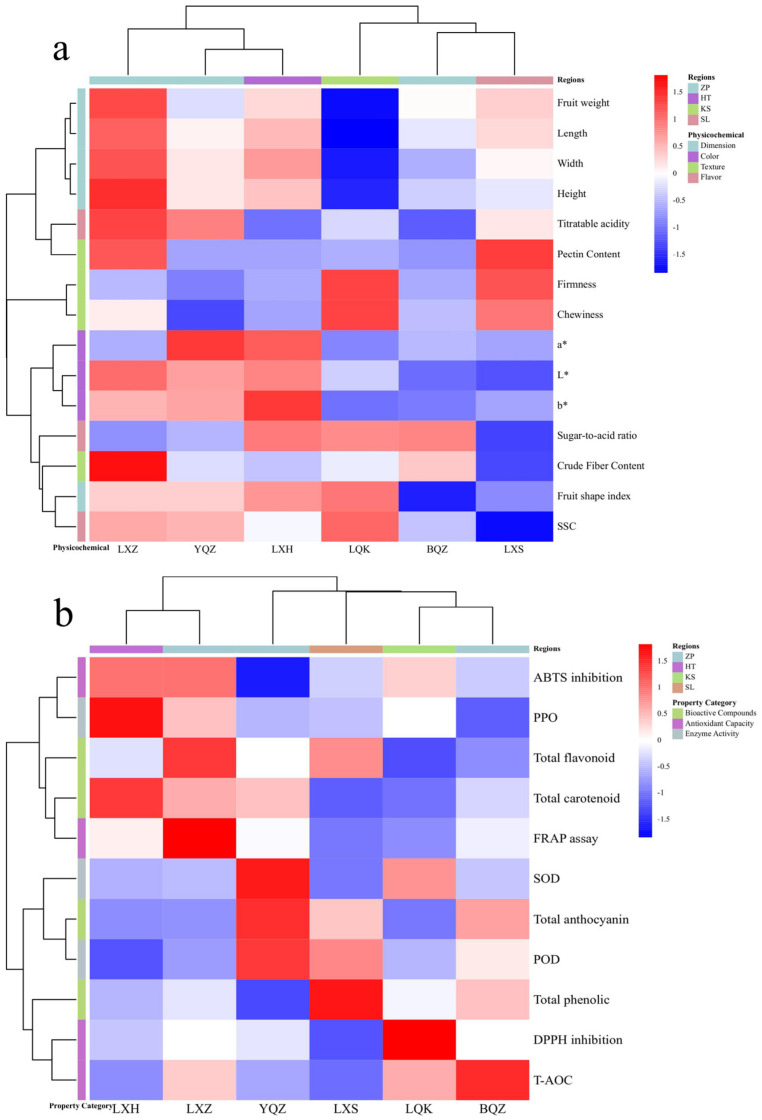
Cluster heatmap analysis of different quince varieties: (**a**) basic physicochemical properties; (**b**) active components and antioxidant activities.

A key finding of this study is the absence of a simple linear correlation between the overall antioxidant efficacy of quince fruits and the content of any individual bioactive component, highlighting the complexity of their antioxidant system (Figure 1a,b). The most direct evidence for this non-linearity comes from the analysis of the relationship between total phenolic content and total antioxidant capacity. Although LXS possessed the highest TPC, its T-AOC was only moderate. Conversely, BQZ exhibited the strongest T-AOC while maintaining a high, yet not the highest, TPC. This discrepancy unequivocally demonstrates that the overall antioxidant capacity of quince arises from a complex synergistic network involving both enzymatic and non-enzymatic components, rather than being dictated by the abundance of any single compound class, such as total phenolics. This finding has important implications for understanding the chemical basis of quince as a functional food. It suggests that in evaluating and selecting quince germplasms with high antioxidant activity or in developing related products, we should move beyond reliance on single metrics and instead focus on the complete profile of antioxidant constituents and their potential synergistic interactions. This provides a novel scientific perspective for developing more precise strategies for food functionality evaluation and product development based on multi-component synergy.

### 3.3. Multivariate Analysis of Multi-Source Sensor Data

#### 3.3.1. Analysis of NIRS

**Figure 2:**
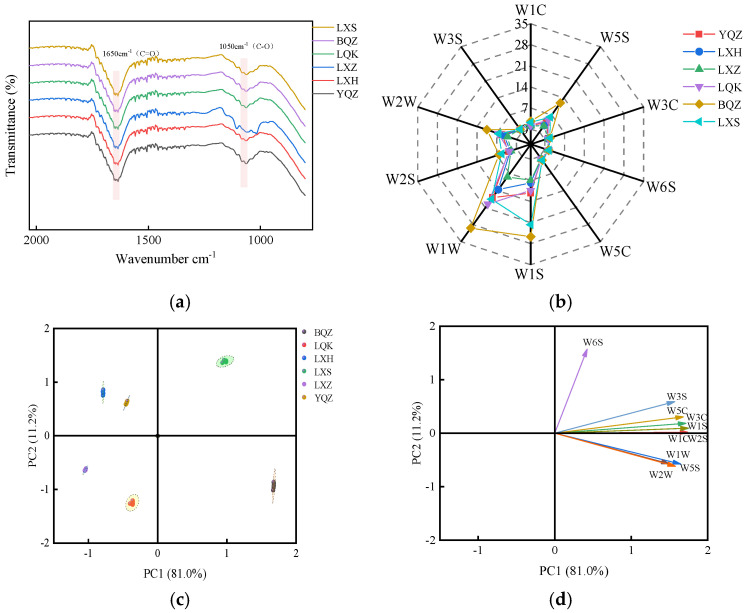
Comprehensive spectral and sensor analysis of different quince varieties: (**a**) Fourier transform mid-infrared (FT-MIR) transmission spectra (Transmittance %) of quince fruits from different accessions. The full spectral scanning range was 400–4000 cm^−1^, and the region 800–2000 cm^−1^ is shown to highlight characteristic absorption peaks (e.g., C=O at ~1630 cm^−1^ and C–O at ~1050 cm^−1^). (**b**) Electronic nose sensor response; (**c**) PCA score plot; (**d**) PCA loading plot.

NIRS revealed cultivar-specific spectral variations (Figure 2a). Characteristic peaks at ~1650 cm^−1^ (C=C stretch) and ~1055 cm^−1^ (C-O stretch) were observed. The intensity at 1650 cm^−1^ correlated with pigment content, while the signal at 1055 cm^−1^ showed a significant positive correlation with firmness (*p* < 0.05), which aligns with the high pectin content and chewiness of LXS.

#### 3.3.2. Electronic Nose Sensor Response and Principal Component Analysis

E-nose analysis detected distinct volatile profiles (Figure 2b). PCA of the sensor data effectively discriminated the geographical origins (Figure 2c), with PC1 and PC2 explaining 92.2% of the variance. The Zepu County cultivars (YQZ, LXZ, BQZ) were distributed along PC1, a gradient later attributed to ester content variation by GC-MS. PC2 separated LXS (aldehyde-rich) from LQK (terpenoid-rich). The loading plot (Figure 2d) identified W1W/W2W (sulfides, terpenoids) as key drivers for PC1, and W5S/W6S (nitrogen oxides, methyls) for PC2.

### 3.4. Composition and Characterization of Volatile Flavor Compounds in Quinces from Different Producing Regions

#### 3.4.1. Analysis of Volatile Compound Content and Variety

**Figure 3:**
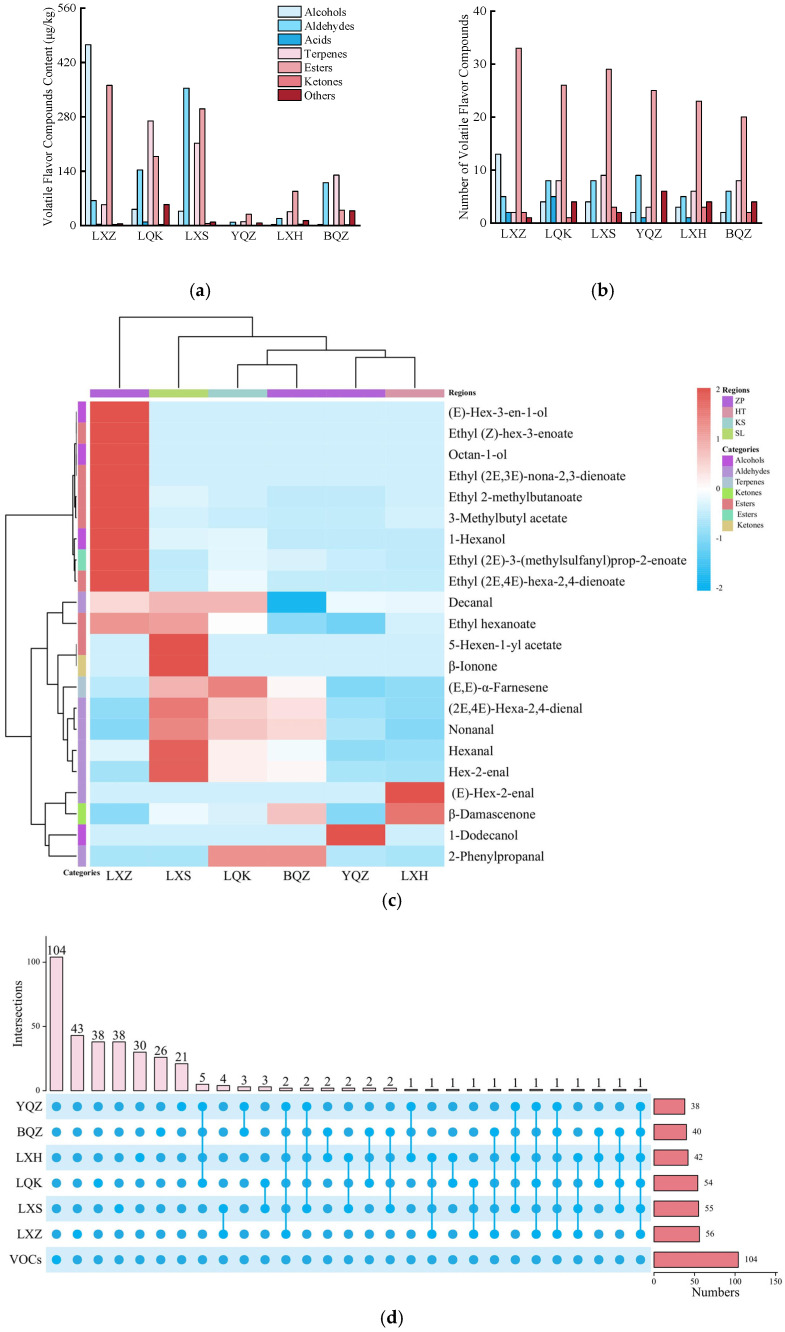
Comprehensive analysis of volatile compounds and characteristic aroma in quince fruits from different regions: (**a**) content of flavor substances, (**b**) number of volatiles, (**c**) OAV cluster heatmap (where red indicates high values and blue indicates low values), and (**d**) Upset plot of aroma-active compounds. The row labeled “VOCs” represents the total library of volatile compounds (union of all sets). Note that the first column (value = 104) represents the union of all compounds across all accessions, not an intersection.

LXZ and LXS were the most abundant sources of volatiles, with total concentrations of 956.36 and 918.32 μg/kg, respectively (Figure 3a,b). Esters were the predominant class, especially in LXZ (361.01 μg/kg). Aldehydes were exceptionally high in LXS (353.83 μg/kg), while LQK was uniquely enriched in terpenoids (269.62 μg/kg). LXZ and LXS also exhibited the most diverse profiles, with 57 species each.

#### 3.4.2. Characterization of Key Aroma Compounds Based on OAV and Cluster Heatmap Analysis

OAV analysis identified 22 key aroma compounds (OAV > 1). Hexanal, nonanal, damascenone, isoamyl acetate, and ethyl hexanoate (OAVs > 50) were established as the most impactful (Figure 3c). The cultivars were segregated into three flavor clusters: an ester-dominant cluster (LXZ, LXS), an aldehyde-ketone cluster (LQK, BQZ), and a cluster with singular potent aromas (LXH with damascenone, OAV = 265.05).

#### 3.4.3. Analysis of Shared and Unique Flavor Compounds Based on Upset Plot

To further elucidate the distribution patterns of flavor compounds across cultivars, an Upset plot was constructed (Figure 3d). For readers less familiar with this visualization, an Upset plot uses a matrix layout in which each row represents a set (e.g., a cultivar), and each column represents a specific intersection of sets. A dot in a cell indicates that the row’s set is included in that column’s intersection. However, in Figure 3d, all dots are cyan, so visual distinction of which specific sets are included in a given column requires close inspection of the dot matrix below each column. The analysis revealed that the entire library of volatile compounds across all cultivars (i.e., the union of all sets, corresponding to the first column of Figure 3d) consisted of 104 compounds, which defines the fundamental aroma background common to Xinjiang quinces and likely represents the characteristic aroma components of this species. According to the horizontal bars on the right, LQK possessed 54 flavor compounds (not the highest number, as LXZ had 56 and LXS had 55), a finding consistent with its distinct flavor profile identified in the OAV analysis (e.g., enrichment of β-ionone). This indicates that LQK has a unique flavor metabolic pathway among the cultivars studied. Additionally, specific shared combinations were observed among certain cultivar groups. A substantial shared combination of 38 compounds was observed between certain cultivar pairs (corresponding to the fourth column of Figure 3d), suggesting conserved flavor metabolism despite significant cultivar and phenotypic differences. Furthermore, a combination of 26 compounds was shared among other specific cultivars (e.g., corresponding to the sixth column of Figure 3d), forming another important secondary core flavor group. This pattern reveals the existence of specific flavor metabolic pathways that operate across different cultivars and geographical origins. 

### 3.5. Screening of Key Differential Markers and Interaction Analysis Based on the OPLS DA Model

To validate the overall discriminative power of the selected markers among the four quince accessions, PLS-DA was first applied to the fused dataset. The PLS-DA model was constructed with the four quince accessions as the categorical Y-variable and the combined spectroscopic and chemical data as the X-matrix. The optimal number of components (5) was determined by 7-fold cross-validation. The model yielded excellent performance: R^2^X(cum) = 0.993, R^2^Y(cum) = 0.998, and Q^2^(cum) = 0.992, with an overall classification accuracy of 100% (Table S1). The permutation test (200 permutations) gave an R^2^ intercept of 0.112 and a Q^2^ intercept of −0.738, confirming no overfitting. These results confirm that the four quince accessions are chemically distinguishable at the overall level.

Subsequently, OPLS-DA was performed for pairwise comparisons between specific accessions to identify accession-specific marker compounds.

#### 3.5.1. OPLS-DA Model Validation and Key Marker Identification for Pairwise Accession Comparison

**Figure 4:**
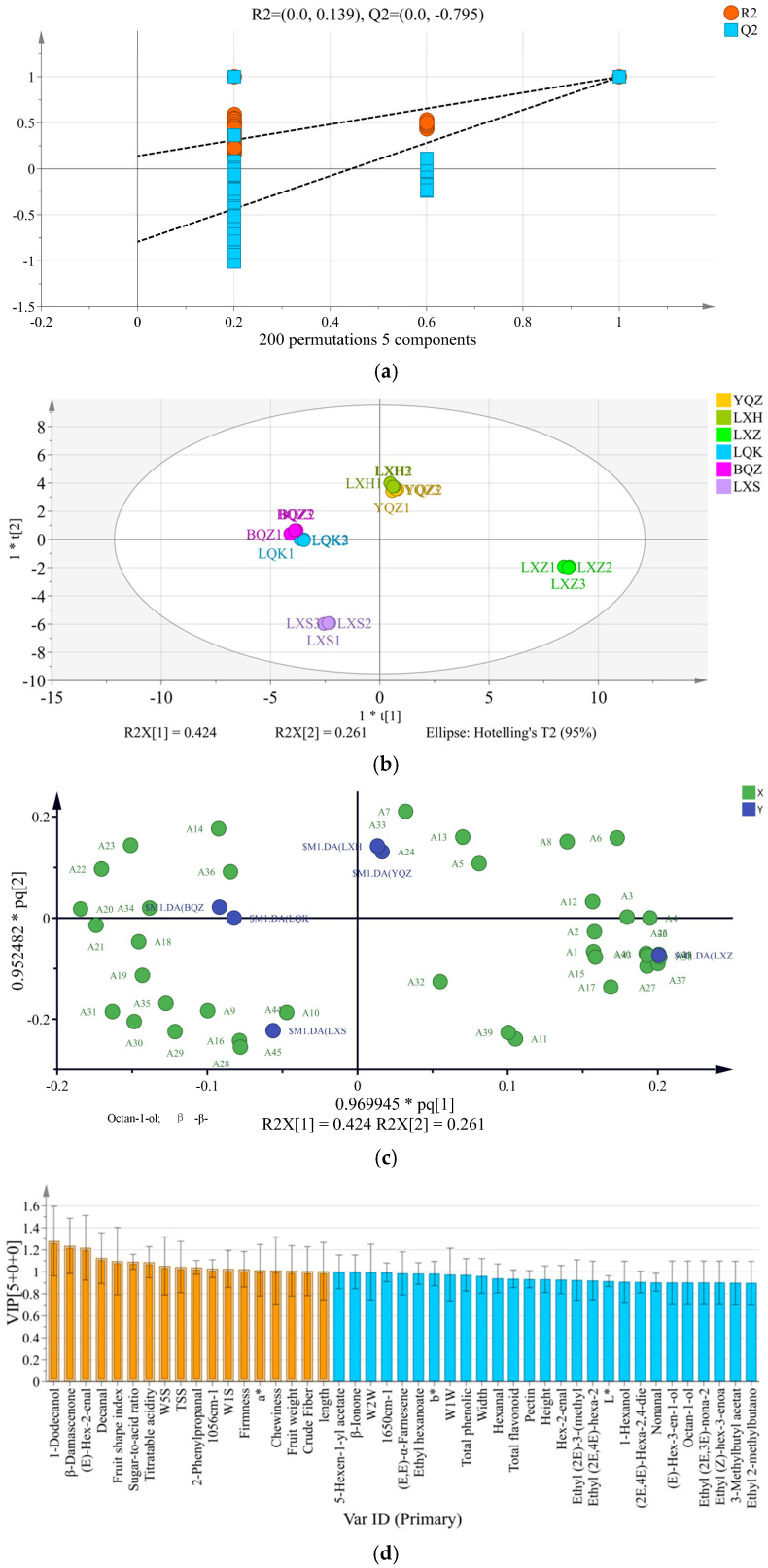

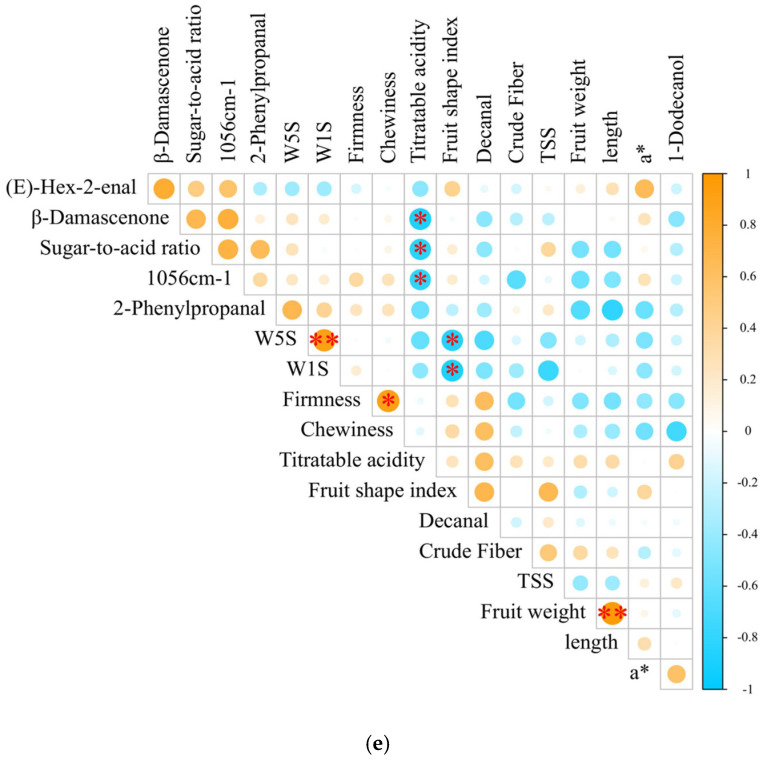
OPLS-DA model for quality evaluation of quince from different regions, key biomarker screening, and correlation analysis. (**a**) OPLS-DA model validation plot based on permutation testing (200 permutations) using 5 predictive components (5+0+0). R^2^ (goodness of fit, orange circles) and Q^2^ (predictive ability, blue squares) are shown. The regression lines and intercepts (left side) assess the model’s robustness and avoid overfitting. (**b**) OPLS-DA score plot showing sample separation according to geographical origin. The x-axis (t[1]) is the predictive component, and the y-axis (t[2]) is the second predictive component. Samples are colored according to variety (e.g., YQZ, LXH, LXZ, LQK, BQZ, LXS) to visualize potential confounding effects between cultivar and origin. The gray ellipse represents the 95% Hotelling’s T^2^ confidence region. (**c**) Loading scatter plot of the OPLS-DA model, showing the contribution of individual variables to the discrimination of the four accessions. Variables farther from the origin have higher discriminatory power. (**d**) Variable Importance in Projection (VIP) value plot. Orange bars indicate VIP > 1 (key discriminant markers); blue bars indicate VIP < 1. (**e**) Correlation heatmap of the 18 key markers. Red indicates positive correlation; blue indicates negative correlation. Asterisks indicate statistical significance: * *p* < 0.05, ** *p* < 0.01). Abbreviations: OPLS-DA, orthogonal partial least squares-discriminant analysis; VIP, variable importance in projection; t[1], predictive component; t[2], predictive component; R^2^, coefficient of determination; Q^2^, predictive ability.

To identify accession-specific marker compounds, OPLS-DA was performed for pairwise comparisons between specific accessions, which is the established application of this method. The model exhibited a strong fit and high predictive ability, as evidenced by the parameters R^2^Y = 0.941 and Q^2^ = 0.759 (Figure 4a). This indicates that the model reliably captured the systematic variance related to geographical origin.

The score plot of the OPLS-DA model (Figure 4b) demonstrated a clear separation among the quince germplasms from different geographical origins along the first predictive component (t[1]). The two principal components, t[1] and t[2], explained 42.4% and 26.1% of the variance in the X-matrix (R^2^X[1] = 0.424, R^2^X[2] = 0.261), respectively. The distinct clustering of samples within the Hotelling’s T2 ellipse (95% confidence interval) visually confirms that the integrated dataset—comprising morphological, physicochemical, spectral, and volatile profiles—contains sufficient information to effectively distinguish germplasms by their origin.

VIP analysis tentatively identified 18 key markers (VIP > 1.0) as core drivers for geographical discrimination, forming a preliminary, multi-dimensional fingerprint (Figure 4d). In the VIP plot, orange bars indicate variables with VIP > 1.0 (key discriminant markers), while blue bars indicate variables with VIP < 1.0 (less important variables). This integrated marker set spans morphological traits (e.g., fruit shape index, weight), physicochemical qualities (e.g., sugar-to-acid ratio, total flavonoids), FT-MIR spectroscopic signatures (e.g., peaks at 1055 and 1650 cm^−1^, which correspond to C–O stretching in carbohydrates and C=O stretching in carbonyl compounds, respectively), key aroma volatiles (e.g., hexanal, β-damascenone, ethyl esters), and electronic nose responses (W1W, W5S). FT-MIR was included because it provides rapid, non-destructive molecular fingerprinting of both volatile and non-volatile components (e.g., sugars, organic acids, phenolic compounds), complementing the volatile-specific analysis of HS-SPME-GC-MS and the olfactory simulation of the E-nose. The inclusion of spectroscopic and volatile markers extends beyond traditional quality metrics, linking macroscopic properties to molecular composition and offering a potential basis for origin traceability. Given the exploratory nature of this study due to the limited sample size (*n* = 18), these findings should be validated in future research with larger, independent sample sets. However, this multi-parameter foundation may support the future development of rapid, non-destructive authentication tools for quince germplasm resources.

#### 3.5.2. Correlation Network Analysis of Key Discriminant Markers

Prior to supervised OPLS-DA, PCA was performed to explore the natural grouping of the samples. The PCA score plot (Figure S2) explained 68.5% of the total variance (PC1: 42.4%, PC2: 26.1%). The plot revealed that samples from different geographical origins were partially separated along the PC1 axis, although some overlap was observed due to the limited sample size and partial confounding between cultivar and origin (e.g., LQK and BQZ clustered closely). This partial separation supported the feasibility of using supervised OPLS-DA to further identify accession-specific discriminant markers.

An OPLS-DA model was built with 5 predictive components (5+0+0) to identify markers responsible for accession discrimination. The model’s validity was rigorously assessed. Figure 4a shows the permutation test (200 permutations, 5 components). The intercepts of the regression lines, directly reported in the plot, are R^2^ = (0.0, 0.139) and Q^2^ = (0.0, −0.795). The low R^2^ intercept (0.139) and the negative Q^2^ intercept (−0.795) confirm the model’s good fit and absence of overfitting.

The score plot (Figure 4b) shows the discrimination of the four accessions along the first two predictive components (t[1] vs. t[2]) of the OPLS-DA model. The four accessions were clearly separated, indicating that the selected chemometric markers effectively discriminated among different quince germplasms.

The Loading Scatter Plot (Figure 4c) was analyzed to visualize the contribution of individual variables to the pairwise discrimination of the four accessions. Variables located farther from the origin in the loading plot exert a stronger influence on the separation between accession pairs. Specifically, 1-Dodecanol and β-Damascenone were positioned at the periphery of the loading space, indicating their dominant roles in discriminating LQK from other accessions. Meanwhile, (E)-Hex-2-enal and Decanal loaded heavily on the opposite quadrant, suggesting their preferential association with LXZ and LXS. Fruit shape index and Sugar-to-acid ratio exhibited intermediate loading positions, reflecting their combined morphological and physicochemical contributions to accession separation. The loading plot thus confirmed that the discriminant markers are distributed across multiple chemical and physical domains, rather than being dominated by a single data type.

To further prioritize the markers, the VIP plot (Figure 4d) was examined. Variables with VIP > 1 were considered key discriminant markers. As shown in Figure 4d, 1-Dodecanol, β-Damascenone, (E)-Hex-2-enal, Decanal, Sugar-to-acid ratio, Fruit shape index, and Titratable acidity were identified as the top contributors to accession discrimination. The consistency between the loading plot and VIP analysis reinforces the reliability of these markers.

To identify the specific discriminating variables, the Loading Scatter Plot (Figure 4d) and VIP plot (Figure 4d) were analyzed. Variables with VIP > 1 were considered key discriminant markers. As shown in Figure 4c, 1-Dodecanol, β-Damascenone, (E)-Hex-2-enal, Decanal, Sugar-to-acid ratio, Fruit shape index, and Titratable acidity were identified as the top contributors to geographical discrimination.

Correlation network analysis (Figure 4e) elucidated the systemic interactions among the 18 key markers identified for accession discrimination. Significant positive correlations were observed among volatile aldehydes and esters, such as between (E)-Hex-2-enal and Decanal, suggesting coordinated biosynthesis from shared lipid precursors. Morphological traits (e.g., Fruit weight) were positively correlated with specific long-chain volatiles, indicating a developmental linkage to flavor compound accumulation. Conversely, a negative correlation was found between the Sugar-to-acid ratio and key aldehydes (e.g., (E)-Hex-2-enal), potentially reflecting a metabolic trade-off between primary and secondary metabolism during ripening.

The network thus reveals that the discriminant markers do not vary independently but form an interconnected system bridging morphology, primary metabolism, and volatile biosynthesis. This integrated correlation structure provides a functional context for the multidimensional chemical fingerprint that effectively discriminates among quince accessions.

## 4. Discussion

As a high-value specialty fruit, quince exhibits quality and flavor profiles that are fundamentally shaped by the interaction between cultivar genetics and geographical origin. This study provides a systematic characterization of the comprehensive quality attributes of different quince cultivars from four major producing regions in Xinjiang. Through integrated analytical approaches and comparison with existing literature, our findings substantially advance the understanding of quality diversity within quince resources.

### 4.1. Morphological and Physicochemical Diversity: Genotypic and Environmental Interplay

The significant morphological and physicochemical variations observed among cultivars suggest the complex interplay between genetic background and growing environment. The fruit weight range (70.78–238.74 g) documented in this study aligns with previous reports on quince [6,26], confirming the substantial natural variation within this species. Fruit size is known to be influenced by the combined effects of genetic background and cultivation environment, including soil fertility, light availability, and water supply [14]. The relatively high single fruit weight of LXZ may be attributed to the more favorable light conditions and irrigation practices in the Zepu production area, which likely promoted photoassimilate accumulation and cell expansion.

Color parameters, crucial for commercial valuation, showed notable diversity. The L value of YQZ (73.20) fell within the range (65.9–74.6) reported for Turkish quince [10], while color variation is primarily regulated by light- and temperature-dependent pigment metabolic pathways involving chlorophyll degradation and the synthesis of carotenoids and anthocyanins [27]. The fruit shape index (0.88–1.01) demonstrated diversity from oblate to nearly round shapes, with LXZ (1.19) exhibiting a typical pyriform morphology. This observation supports the view emphasized in [28] that genetic factors are the predominant source of fruit phenotypic differentiation.

Regarding flavor-related traits, sugar-acid composition showed marked cultivar specificity. LQK exhibited high SSC (14.90%) while LXZ showed elevated titratable acidity (3.52 g/L) [29]. These significant inter-cultivar differences are consistent with observations on ‘Limon’ and ‘Ekmek’ type quinces, as well as with reports by Hanci et al. [10] on high-acidity Turkish cultivars. The SSC range (9.63–14.90%) overlaps with values reported in previous studies [9,30,31]. The sugar-to-acid ratio critically determined the sweet-sour balance, with high ratios (>5.7) in LXH, LQK, and BQZ favoring fresh consumption, while lower ratios in LXZ and LXS indicated better processing suitability—confirming the ratio’s established impact on sensory perception [26].

Our findings further indicate quince as a significant source of functional components. The pectin content in LXS (1.86%) approximated levels reported by Israa et al. [32] and exceeded values from Shah et al. [33] and Sharma et al. [34]. LXZ contained exceptional crude fiber (5.86%), substantially surpassing concentrations in apple [35] and pear [36], though exceeding the range observed by Kostecka-Gugala [4]. Correlation analysis revealed significant relationships between pectin and moisture content, and between crude fiber and firmness, reinforcing the established associations between cell wall components and textural properties in Rosaceae fruits [37].

### 4.2. Bioactive Components and Antioxidant Synergism

The phytochemical profiling confirmed quince’s rich bioactive composition. Total phenolic content (39.20–143.40 mg/100 g) encompassed the ranges reported by Anber et al. [28] and Shah et al. [33]. Carotenoid content (0.47–1.89 mg/g) corresponded with reports by Katarzyna et al. [3] and Hamauzu et al. [5], reflecting known influences of genetic background, maturity stage, and growing conditions [6].

A fundamental finding concerns the non-linear relationship between individual components and antioxidant efficacy. Despite LXS possessing the highest polyphenol content, BQZ demonstrated superior T-AOC, suggesting that antioxidant effects may derive from potential synergistic associations among multiple components rather than single compounds. The methodological variations observed across DPPH, ABTS, and FRAP assays reflect their differential sensitivity to hydrophobic and hydrophilic antioxidants, necessitating comprehensive multi-method evaluation. The absence of direct correlations between enzymatic (PPO, POD, SOD) and non-enzymatic antioxidant pools indicates that the antioxidant system likely constitutes a complex complementary defense system, rather than a single metabolic pathway.

### 4.3. Volatile Profiling and Aroma Signatures

Volatile characterization revealed substantial aromatic complexity, with 105 identified compounds, exceeding the diversity reported by Tomasino et al. [38]. The predominance of esters, aldehydes, and alcohols aligns with established quince volatile profiles [9,39].

OAV analysis identified hexanal, nonanal, damascenone, isoamyl acetate, and ethyl hexanoate as primary aroma contributors. The exceptional OAV of damascenone in LXH (265.05) suggests strong geographical specificity, consistent with its established origin from carotenoid degradation [40]. The identification of characteristic compounds including α-farnesene and ethyl octanoate corroborates findings by Rather et al. [9]. The consensus regarding key volatiles reinforces their significance, while ester contributions to fruity aromas align with observations in Chinese quince [41]. The distinct combinatorial patterns of these aroma-active compounds provide a chemical basis for cultivar discrimination and geographical traceability.

### 4.4. Advanced Analytical Integration for Quality Assessment

The integration of spectroscopic and chemometric approaches established a comprehensive quality evaluation framework. FT-MIR spectroscopy successfully correlated specific absorption bands with quality parameters, with 1650 cm^−1^ (C=C) and 1055 cm^−1^ (C-O) peaks associated with pigments and cell wall components, respectively, indicating the potential of FT-MIR for non-destructive assessment as demonstrated in apple studies [23].

Multivariate analysis effectively decoded complex quality data. The OPLS-DA model was built with five predictive components (5+0+0). The model’s validity was confirmed by a permutation test (200 permutations), which yielded a low R^2^ intercept (0.139) and a negative Q^2^ intercept (−0.795), demonstrating no overfitting despite the higher component count. The model identified 18 key markers (VIP > 1) for accession discrimination, with 1-Dodecanol, β-Damascenone, (E)-Hex-2-enal, Decanal, and the Sugar-to-acid ratio being the most significant contributors.

In accession traceability studies, the potential confounding effect of variety and geographic origin must be carefully considered. In this study, different accessions were sampled from distinct geographical origins, meaning that varietal and site effects are not fully separable. However, previous studies have demonstrated that genotype exerts a dominant influence on fruit chemical composition compared with growing environment. Wojdyło et al. [42] analyzed 13 quince varieties and 5 genotypes and found that total polyphenol content varied more than two-fold across genotypes (from 1709.43 to 3436.56 mg/100 g dry weight), demonstrating that genetic background exerts a profound influence on quince phytochemical profiles. Rop et al. [43] reported significant differences in basic chemical characteristics among 22 quince genotypes and cultivars, further confirming the substantial genetic variability within quince germplasm. In a related Rosaceae species, Harnly et al. [44] systematically evaluated the impact of genetics and environment on cranberry fruit metabolites using 15 genotypes grown across 16 locations; their multi-factorial multivariate analysis revealed that genotype was a statistically significant (*p* < 0.001) determinant of metabolite composition. Consistent with these findings, the primary objective of the present study was to establish a chemometric framework for accession-level quality evaluation, rather than to partition genotype by environment interactions. For practical germplasm characterization purposes, the observed accession-level clusters remain meaningful, as they reflect the integrated chemical phenotype of each germplasm under its native cultivation conditions. Nonetheless, we acknowledge that analyzing the same varieties across all origins would be the gold standard. Therefore, the current findings, while statistically robust, are primarily exploratory and should be validated in future studies with independent sample sets that include common varieties across multiple origins.

Marker-environment relationships were evident, particularly the association between high damascenone in LXH and carotenoid degradation under intense light/drought stress, consistent with drought-induced norisoprenoid accumulation mechanisms [3]. This multivariate analysis goes beyond simple visualization: it provides a validated statistical framework to decode the complex metabolite network (Figure 4e), enabling the identification of key discriminant markers and their systemic interactions. The identification of three distinct antioxidant phenotypes provides preliminary insights into environmental adaptation strategies within Rosaceae species.

### 4.5. Limitations of the Study

Several limitations of this study should be acknowledged. First, regarding study design and sampling: The sample size was limited to six accessions with three biological replicates each (*n* = 18), which restricted the statistical power of the multivariate models. More importantly, due to the sampling design, cultivar (accession) and geographical origin were partially confounded: as shown in Table 1, some origins (e.g., Hotan, Kashi, Shule) are represented by only one variety, while Zepu County contributed three accessions (LXH, LXS, LQK). Consequently, the OPLS-DA model cannot fully separate cultivar-specific effects from geographical effects. We explicitly acknowledge this as a major limitation of the current study. Although a subgroup analysis using only the ‘apple-shaped’ varieties (LXH, LXS, LQK) was considered to partially address this, the limited sample size of this subgroup (*n* = 9) rendered such an analysis statistically unreliable. Nevertheless, as discussed in Section 4.4, previous studies have established that genotypic differences are the primary driver of chemical variations in quince and related Rosaceae species [42,43,44]. The primary goal of this study was to develop a chemometric framework for accession level quality evaluation, and the observed clusters remain meaningful for germplasm characterization purposes, as they reflect the integrated phenotypic performance of each germplasm under its native conditions. Future studies with multi-site common-garden experiments would be valuable to formally separate genetic and environmental contributions.

Second, regarding volatile compound identification: A methodological limitation is that retention indices (RI) were not calculated due to the absence of n-alkane standards. As a result, compound identification relied primarily on mass spectral matching with the NIST 17 and NIST 18 libraries (similarity score > 80%). This increases the risk of misidentifying structurally similar compounds, particularly positional or geometric isomers. Future studies should include alkane standards to enable RI calculation and further validation.

Third, regarding statistical modeling and marker selection: The high R^2^Y (0.941) and Q^2^ (−0.795) values should be interpreted with caution given the small sample size and the large number of variables, which collectively increase the risk of overfitting. The model presented here is therefore exploratory and provides a preliminary framework for quality assessment of Xinjiang quince. Validation with larger, independent sample sets is essential before any practical application. Additionally, the selection of key discriminant markers was based solely on VIP > 1.0, a statistical criterion that does not guarantee biological relevance. Therefore, the biological significance of the 18 identified markers should be interpreted with caution. Future studies should integrate additional validation metrics such as ANOVA or pathway enrichment analysis to strengthen marker interpretation.

Despite these limitations, the multi-analytical approach developed in this study offers a foundation for future research on quince germplasm characterization and quality evaluation.

## 5. Conclusions

This study systematically deciphered the quality and flavor diversity of major quince cultivars from Xinjiang, China, by integrating multi-source spectroscopy and chemometrics. The findings revealed significant chemodiversity among the accessions, with distinct cultivars exhibiting targeted potential: LXZ (high acidity and esters) is optimal for fruit wine; LXS (rich in pectin and polyphenols) is promising for functional foods; and LXH (abundant in damascenone) serves as a premium resource for flavorings. The combination of FT-MIR, E-nose, and HS-SPME-GC-MS/OAV, supported by multivariate analysis, appeared to be an effective strategy for the rapid assessment and accession-level discrimination. PLS-DA validation confirmed the overall discriminability among the four accessions with 100% accuracy, while OPLS-DA pairwise comparisons identified accession-specific marker compounds. Notably, correlation network analysis suggested significant associations between non-volatile precursors and key aroma compounds, offering preliminary insights into the potential metabolic basis of flavor formation. However, due to the limited sample size (*n* = 18) and partial confounding between cultivar and origin, these findings should be considered exploratory. Nevertheless, consistent with previous studies demonstrating that genotypic differences are the primary driver of chemical variations in quince and related Rosaceae species, the observed accession-level classification remains practically relevant for germplasm characterization. Overall, this work provides a preliminary methodological and theoretical foundation for the authenticity verification, germplasm characterization, and targeted high-value processing of Xinjiang quince. Future research employing integrated multi-site common-garden experiments is recommended to further investigate the molecular basis underlying these critical quality traits and to formally separate genetic and environmental contributions.

## Figures and Tables

**Table 1:** Appearance and basic physicochemical indices of different quince varieties.

Indicator	YQZ	LXH	LXZ	LQK	BQZ	LXS
Fruit weight (g)	154.20 ^e^	182.40 ^c^	238.74 ^a^	70.78 ^f^	167.93 ^d^	185.92 ^b^
Longitudinal (mm)	75.23 ± 1.23 ^d^	81.66 ± 0.58 ^b^	91.20 ± 2.37 ^a^	45.79 ± 1.61 ^f^	71.08 ± 2.08 ^e^	78.21 ± 1.56 ^c^
Transverse (mm)	73.41 ± 1.24 ^d^	81.73 ± 2.14 ^b^	89.75 ± 1.60 ^a^	46.04 ± 2.07 ^f^	62.52 ± 2.12 ^e^	72.03 ± 1.34 ^c^
Height (mm)	78.30 ± 2.13 ^c^	84.49 ± 1.72 ^b^	108.16 ± 3.01 ^a^	39.87 ± 1.27 ^f^	66.75 ± 1.25 ^e^	71.22 ± 2.64 ^d^
Fruit shape index	0.98 ± 0.02 ^b^	1.00 ± 0.02 ^a^	0.98 ± 0.01 ^b^	1.01 ± 0.03 ^a^	0.88 ± 0.02 ^d^	0.92 ± 0.02 ^c^
*L* ^*^	73.20 ± 1.80 ^c^	77.14 ± 1.62 ^b^	80.73 ± 1.63 ^a^	51.13 ± 1.44 ^d^	35.73 ± 2.08 ^e^	32.28 ± 0.79 ^f^
*a* ^*^	7.21 ± 1.06 ^a^	6.58 ± 0.54 ^b^	2.58 ± 0.45 ^d^	1.91 ± 0.13 ^f^	2.70 ± 1.30 ^c^	2.39 ± 0.61 ^e^
*b* ^*^	34.31 ± 2.00 ^b^	44.22 ± 1.34 ^a^	33.05 ± 0.95 ^c^	13.70 ± 0.68 ^f^	14.20 ± 1.42 ^e^	18.05 ± 0.90 ^d^
Firmness (N)	427.26 ± 18.55 ^e^	459.36 ± 46.47 ^d^	469.29 ± 26.51 ^c^	672.01 ± 2.73 ^a^	459.43 ± 11.93 ^d^	662.98 ± 12.33 ^b^
Chewiness (gf)	50.82 ± 49.81 ^f^	115.58 ± 125.74 ^e^	192.99 ± 14.40 ^c^	314.09 ± 41.51 ^a^	136.02 ± 16.65 ^d^	279.07 ± 25.07 ^b^
SSC (%)	13.87 ± 0.51 ^c^	12.77 ± 0.64 ^d^	13.97 ± 0.38 ^b^	14.90 ± 0.41 ^a^	12.07 ± 0.63 ^e^	9.63 ± 0.27 ^f^
Sugar-to-acid ratio	4.24 ± 0.15 ^d^	5.85 ± 0.11 ^a^	3.97 ± 0.17 ^e^	5.73 ± 0.13 ^c^	5.78 ± 0.16 ^b^	3.38 ± 0.13 ^f^
Titratable acidity (g/L)	3.27 ± 0.11 ^b^	2.18 ± 0.08 ^e^	3.52 ± 0.13 ^a^	2.60 ± 0.11 ^d^	2.09 ± 0.14 ^f^	2.85 ± 0.07 ^c^
Pectin content (%)	0.57 ± 0.02 ^d^	0.58 ± 0.03 ^d^	1.73 ± 0.05 ^b^	0.63 ± 0.04 ^c^	0.51 ± 0.02 ^e^	1.86 ± 0.04 ^a^
Crude fiber content (%)	3.90 ± 0.21 ^c^	3.70 ± 0.19 ^d^	5.86 ± 0.16 ^a^	3.99 ± 0.11 ^e^	4.50 ± 0.17 ^b^	2.82 ± 0.15 ^f^

Note: Values are expressed as mean ± standard deviation (*n* = 3). Different lowercase letters in the same column indicate significant differences at *p* < 0.05.

**Table 2:** Active components and antioxidant activities of different quince varieties.

Indicator	YQZ	LXH	LXZ	LQK	BQZ	LXS
Total phenolic (mg GAE/100 g)	39.20 ± 1.25 ^f^	65.40 ± 2.07 ^e^	78.10 ± 2.31 ^d^	81.40 ± 1.81 ^c^	100.30 ± 2.06 ^b^	143.40 ± 3.35 ^a^
Total flavonoid (mg RE/100 g)	73.85 ± 2.14 ^c^	68.60 ± 1.82 ^d^	101.25 ± 2.64 ^a^	48.55 ± 2.02 ^f^	57.10 ± 1.63 ^e^	89.80 ± 2.57 ^b^
Total anthocyanin (mg/100 g)	1.93 ± 0.11 ^a^	1.26 ± 0.08 ^e^	1.27 ± 0.12 ^d^	1.22 ± 0.07 ^f^	1.70 ± 0.13 ^b^	1.62 ± 0.11 ^c^
Total carotenoid (mg/g)	1.37 ± 0.07 ^c^	1.89 ± 0.05 ^a^	1.46 ± 0.04 ^b^	0.56 ± 0.04 ^e^	0.95 ± 0.03 ^d^	0.47 ± 0.03 ^f^
ABTS scavenging capacity (%)	41.80 ± 1.37 ^e^	52.00 ± 2.03 ^a^	52.03 ± 1.42 ^a^	49.40 ± 1.61 ^b^	46.61 ± 2.36 ^d^	46.74 ± 1.62 ^c^
DPPH scavenging capacity (%)	22.50 ± 0.96 ^c^	21.28 ± 0.81 ^d^	23.61 ± 1.04 ^b^	32.60 ± 1.23 ^a^	23.50 ± 0.67 ^b^	17.34 ± 1.06 ^e^
FRAP assay	0.29 ± 0.01 ^c^	0.33 ± 0.02 ^b^	0.68 ± 0.04 ^a^	0.13 ± 0.02 ^e^	0.27 ± 0.03 ^d^	0.10 ± 0.01 ^f^
PPO (U/g)	12.12 ± 1.02 ^e^	31.35 ± 0.95 ^a^	20.64 ± 1.14 ^b^	16.62 ± 0.87 ^c^	6.78 ± 0.65 ^f^	12.66 ± 1.06 ^d^
POD (U/g)	1523.90 ± 10.34 ^a^	124.95 ± 4.31 ^f^	394.45 ± 7.64 ^e^	492.45 ± 5.13 ^d^	860.00 ± 11.03 ^c^	1220.10 ± 12.42 ^b^
SOD (U/g)	29.27 ± 1.02 ^a^	6.62 ± 0.65 ^e^	7.17 ± 0.32 ^d^	20.61 ± 0.83 ^b^	8.26 ± 0.64 ^c^	2.52 ± 0.73 ^f^
T-AOC (μmol/g)	1.03 ± 0.05 ^d^	0.79 ± 0.06 ^e^	2.24 ± 0.06 ^c^	2.50 ± 0.03 ^b^	3.63 ± 0.07 ^a^	0.56 ± 0.05 ^f^

Note: Values are expressed as mean ± standard deviation (*n* = 3). Different lowercase letters in the same column indicate significant differences at *p* < 0.05.

## Data Availability

The original contributions presented in this study are included in the article/Supplementary Material. Further inquiries can be directed to the corresponding author.

## Supplementary Materials

The following supporting information can be downloaded at: https://www.mdpi.com/article/10.3390/foods15142558/s1, Table S1: A complete list of volatile components. Table S2: Correspondence between the code numbers (A1–A45) and volatile components; Figure S1: A map illustrating the geographical locations of the sampling sites and PCA. Figure S2: The results of PLS-DA.

## Abbreviations

The following abbreviations are used in this manuscript:

BQZ	*Cydonia oblonga* var. lusitanica (large-fruited quince from Zepu County)
LQK	*Cydonia oblonga* var. Pyramidalis (pyramidalis quince from Kashi City)
LXH	*Cydonia oblonga* var. maliformis (apple-shaped quince from Hotan City)
LXZ	*Cydonia oblonga* var. Maliformis (apple-shaped quince from Zepu County)
YQZ	*Cydonia oblonga* var. pyriformis (pear-shaped quince from Zepu County)
SSC	Soluble solid content
DPPH	2,2-Diphenyl-1-picrylhydrazyl
FRAP	Ferric reducing antioxidant power
T-AOC	Total antioxidant capacity
HS-SPME-GC-MS	Headspace solid-phase microextraction gas chromatography-mass spectrometry
VIP	Variable importance in projection
OAV	Odor activity value
