# Supplementary material for: Chemometrics Combined with Multi-Source Spectroscopy for Fruit Germplasm Quality Evaluation: A Case Study on Quince (Cydonia oblonga)
Source: Foods. 2026 Jul 21;15(14):2558. doi: 10.3390/foods15142558 (PMC13408273; doi:10.3390/foods15142558)

Supplementary Table S1

Table 1: Content of volatile flavor compounds in quince from different origins/varieties (µg/kg)

Volatile Flavor Compounds	Relative Content（µg/kg）						Odor Descriptors
	YQZ	LXH	LXZ	LQK	BQZ	LXS	
Lauryl alcohol	0.17	-	-	-	-	-	Fatty aroma, honey aroma
2-Hexyl-1-decanol	0.08	-	0.91	-	-	-	Fatty aroma, waxy aroma
cis-6,cis-9-Pentadecadien-1-ol	-	0.41	-	-	-	-	Green aroma, fruity aroma
S-2-Methyl-1-butanol	-	1.13	-	-	-	-	Apple, pear
6-Methyl-5-hepten-2-ol	-	-	1.71	-	-	-	Fruity aroma
trans-3-Hexenol	-	-	78.97	-	-	-	Fruity-sweet aroma
2-Nonanol	-	-	1.90	-	-	-	Fatty aroma, waxy aroma
5-Methyl-2-hexanol	-	-	7.53	-	-	-	Fruity aroma, sweet aroma
1-Pentanol	-	-	0.71	-	-	-	Waxy taste
1-Octanol	-	-	2.29	-	-	-	Citrus, rose
1-Nonanol	-	-	0.84	-	-	-	Green grass taste
1-Hexanol	-	-	361.97	37.55	-	33.25	Sweet aroma, fruity aroma
1-Heptanol	-	-	1.01	-	-	-	Green note
3-Methyl-1-butanol	-	-	0.88	-	-	-	Banana, green grass
2-Methyl-1-butanol	-	-	5.47	-	-	-	Wine taste, banana
trans-Farnesol	-	-	-	1.79	-	-	Floral aroma, sweet aroma
trans-2-Undecen-1-ol	-	-	-	0.87	-	-	Green aroma, fatty aroma
Phytol	-	-	-	1.55	-	-	Green aroma, floral aroma
β-Acorinol	-	-	-	-	1.08	-	Woody aroma, floral aroma
trans-2-Decen-1-ol	-	-	-	-	-	1.09	Citrus, floral aroma
trans-Geranylgeraniol	-	-	-	-	-	1.43	Marigold flower aroma
Phytol	-	1.09	1.39	-	1.55	1.52	Floral aroma, sweet aroma
Alcohols	0.25	2.62	465.57	41.75	2.62	37.28	
Hexanal	5.83	12.62	59.25	101.09	75.53	254.94	Grass taste, wine
2-Hexenal	1.07	-	-	31.15	28.53	87.43	Green grass taste
trans,trans-2,4-Hexadienal	0.17	-	-	1.79	1.55	3.11	Green grass, fried note
Phenylacetaldehyde	0.27	0.08	0.40	3.10	0.12	0.91	Honey-sweet floral aroma
Nonanal	0.70	-	-	3.22	2.84	4.66	Citrus, fat, floral aroma
Decanal	0.45	0.44	0.61	0.72	-	0.72	Fatty taste, floral aroma, green grass
Undecanal	0.08	-	-	0.25	-	0.36	Fatty aroma, waxy aroma
Dodecanal	0.19	0.72	-	-	-	-	Orange aroma, lemon aroma
trans-2-Hexenal	-	4.61	-	-	-	-	Almond, green grass
3-Methylbutanal	-	-	3.41	-	-	-	Almond, cheese
Benzaldehyde	-	-	0.73	-	-	-	Almond, bitter almond
trans-2-Heptenal	-	-	-	-	-	1.70	Fatty aroma, fruity aroma
α-Methylphenylacetaldehyde	0.05	-	-	1.81	1.83	-	Floral aroma, hyacinth, lily of the valley
Aldehydes	8.82	18.46	64.42	143.14	110.4	353.83	
Caprylic acid	0.46	0.51	-	0.75	-	-	Cheese, pineapple, coconut

Myristic acid	-	-	2.95	-	-	-	Fatty aroma
Pentadecanoic acid	-	-	0.86	0.74	-	-	Fatty aroma, waxy aroma
Palmitic acid	-	-	-	5.49	-	-	Fatty aroma
Oleic acid	-	-	-	1.53	-	-	
Stearic acid	-	-	-	0.80	-	-	Waxy aroma
Acids	0.46	0.51	3.80	9.30	-	-	
α-Farnesene	8.72	20.63	52.90	235.75	109.95	183.15	Jasmine
Berganotene	0.33	1.35	-	15.69	6.69	11.74	Bergenia aroma, citrus aroma
Damascenone	1.04	13.25	1.17	4.02	8.86	4.77	Apple, honey, rose
trans-β-Farnesene	-	0.20	-	1.66	0.66	0.92	Floral aroma, fatty aroma
γ-Muurolene	-	0.04	-	2.48	0.83	3.54	Woody aroma, essential oil aroma
Octahydro-6-methyl-3-methylenebenzofuran	-	0.50	-	0.61	1.03	-	Sweet aroma, floral aroma
Geranyl-α-terpinene	-	-	-	3.89	1.29	2.27	Floral aroma, citrus aroma
2,3-Dimethyl-1-pentene	-	-	-	0.96	-	1.63	Green aroma, fatty aroma
trans-6-Methyl-3-undecene	-	-	-	-	0.72	-	Orange peel, green aroma
2,3,6-Trimethyl-1,5-heptadiene	-	-	-	-	-	2.35	Fatty aroma, green aroma
Isoprene	-	-	-	4.56	-	1.87	Turpentine aroma
Terpenes	10.09	35.98	54.07	269.62	130.03	212.24	
cis-9-Pentadecenoic acid ethyl ester	0.89	-	1.16	3.30	1.04	1.02	Fruity aroma, waxy aroma
trans-2-Butenoic acid ethyl ester	0.18	0.72	49.00	-	-	-	Apple, pear
2-Methylbutyric acid ethyl ester	0.26	2.00	36.72	1.53	-	3.58	Apple, banana, sweet aroma
cis-3-Hexenyl formate	1.63	-	-	-	-	-	Green aroma, green apple aroma
2-Methyl-1-butanol acetate	0.19	1.82	-	3.71	0.07	6.05	Banana, pineapple
Isoamyl acetate	1.43	4.31	85.30	2.61	1.36	4.79	Banana, fruit, sweet aroma
Hexanoic acid ethyl ester	2.59	26.41	72.74	36.40	9.58	68.82	Apple, green grass, orange peel taste
Hexanoic acid hexyl ester	0.09	2.14	1.46	2.71	1.79	12.02	Apple aroma
2-Hexenoic acid ethyl ester	0.07	0.44	5.70	0.67	-	1.33	Green aroma, fruity aroma
6-Heptenoic acid ethyl ester	0.10	0.38	0.39	0.53	-	1.52	Green aroma
Heptanoic acid ethyl ester	0.61	3.11	14.39	6.32	1.06	14.25	Pineapple, fruit
3-Hydroxyhexanoic acid ethyl ester	1.26	0.68	3.03	2.03	1.24	2.28	Sweet aroma, milk aroma
trans-3-Methylthio-2-propenoic acid ethyl ester	0.45	-	14.48	1.58	1.15	-	Sulfur aroma, pungent aroma
7-Octenoic acid ethyl ester	2.64	7.74	5.76	10.65	2.24	19.65	Green aroma, fruity aroma
Octanoic acid ethyl ester	11.37	26.53	26.09	82.08	9.53	116.09	Lily, pear, floral aroma
2-Methylbutyric acid hexyl ester	0.18	-	-	2.23	0.44	7.83	Pineapple, banana
trans-2-Octenoic acid ethyl ester	0.43	1.25	5.66	2.78	0.81	2.76	Green cucumber, green tomato
Nonanoic acid ethyl ester	0.27	-	0.37	0.43	-	0.75	Fruity aroma
3-Hydroxyoctadecanoic acid ethyl ester	0.53	1.42	0.97	-	1.64	-	Fatty aroma, sweet aroma
trans-4-Decenoic acid ethyl ester	0.40	0.42	0.71	2.07	1.24	3.47	Fatty aroma, fruity aroma
9-Decenoic acid ethyl ester	0.55	1.06	0.49	2.47	-	2.45	Cream aroma, fruity aroma
Decanoic acid ethyl ester	1.30	1.94	2.74	6.63	1.07	3.53	Apple, grape
Lauric acid ethyl ester	0.76	1.76	2.21	-	-	-	Floral aroma, sweet aroma
Myristic acid ethyl ester	0.43	-	1.07	3.25	1.79	2.05	Fruity aroma
Palmitic acid ethyl ester	0.47	0.60	0.93	1.58	1.89	1.03	Apple, pineapple
4-Hexen-1-ol acetate	-	2.80	-	-	-	8.24	Green aroma, fruity aroma

Benzoic acid ethyl ester	-	0.37	2.27	0.44	1.23	-	Cherry, grape aroma
cis-3-Hexenyl isovalerate	-	0.20	-	0.26	0.14	1.31	Strawberry, green aroma
Octanoic acid methyl ester	-	0.04	0.18	0.43	-	2.12	Apple, fruity aroma
Propanoic acid ethyl ester	-	-	3.52	-	-	-	Pineapple, apple
2-Methylpropanoic acid ethyl ester	-	-	1.09	0.38	0.54	-	Banana
3-Hydroxypentanoic acid ethyl ester	-	-	0.77	-	-	-	Fruity aroma
2,3-Nonadienoic acid ethyl ester	-	-	1.18	-	-	-	Cucumber aroma, green aroma
3-Hydroxybutanoic acid ethyl ester	-	-	9.94	-	-	-	Milk aroma, fruity aroma
3-Hexenoic acid ethyl ester	-	-	2.68	-	-	-	Green aroma, fruity aroma
trans-2-Butenoic acid methyl ester	-	-	1.66	-	-	-	Apple, pear, green aroma
2,4-Hexadienoic acid ethyl ester	-	-	6.33	0.89	-	-	Fatty aroma
5-Hexen-1-ol acetate	-	-	-	-	-	5.22	Green aroma, fruity aroma
Butanoic acid hexyl ester	-	-	-	-	-	0.96	Pineapple, fruity aroma
5-Hexen-1-yl propionate	-	-	-	-	-	1.35	Green apple, green aroma
Angelic acid ethyl ester	-	-	-	-	-	2.85	Apple, fatty aroma
Pentyl acetate	-	-	-	-	-	2.09	Banana, pear
Heptyl acetate	-	-	-	-	-	1.18	Grape, citrus
Esters	29.07	88.16	361.01	177.96	39.83	300.57	
2-Nonadecanone	-	0.24	0.57	-	-	-	Sweet aroma, fatty aroma
Geranylacetone	-	2.06	2.29	2.91	2.01	2.29	Citrus aroma, woody aroma
β-Ionone	-	-	-	-	-	1.75	Violet aroma, fruity aroma
α-Ionone	-	1.23	-	-	0.33	1.29	Violet aroma, woody aroma
Ketones	-	3.52	2.87	2.91	2.34	5.33	
3,4-Dimethyl-1-hexene	0.47	-	-	-	-	-	Green aroma, fatty aroma
Methoxyphenyl oxide	0.64	2.26	-	3.58	3.03	-	Woody aroma
p-Cymene	0.10	-	-	1.21	1.57	-	Citrus, floral aroma
Indoline	2.09	5.25	-	5.98	9.88	7.03	Sweet aroma, floral aroma
Azulene	1.81	1.43	-	-	-	2.04	-
1,3-Diisopropyl-1,3-cyclopentadiene	1.74	4.07	4.63	43.38	23.59	-	Fatty aroma
Others	6.85	13.00	4.63	54.15	38.08	9.07	

Note: Values are expressed as mean ± standard deviation (n = 3).

Supplementary Table S2

Fruit weight	A1	1-Dodecanol	A24
Fruit length	A2	(E)-Hex-3-en-1-ol	A25
Width	A3	Octan-1-ol	A26
Height	A4	1-Hexanol	A27
Fruit shape index	A5	Hexanal	A28
L*	A6	Hex-2-enal	A29
a*	A7	(2E,4E)-Hexa-2,4-dienal	A30
b*	A8	Nonanal	A31
Firmness	A9	Decanal	A32
Chewiness	A10	(E)-Hex-2-enal	A33
Pectin Content	A11	2-Phenylpropanal	A34
Crude Fiber Content	A12	(E,E)-α-Farnesene	A35
TSS	A13	β-Damascenone	A36
Sugar-to-acid ratio	A14	Ethyl 2-methylbutanoate	A37
Titratable acidity	A15	3-Methylbutyl acetate	A38
Total phenolic	A16	Ethyl hexanoate	A39
Total flavonoid	A17	Ethyl (2E)-3-(methylsulfanyl)prop-2-enoate	A40
W5S	A18	Ethyl (2E,3E)-nona-2,3-dienoate	A41
W1S	A19	Ethyl (Z)-hex-3-enoate	A42
W1W	A20	Ethyl (2E,4E)-hexa-2,4-dienoate	A43
W2W	A21	5-Hexen-1-yl acetate	A44
1056cm-1	A22	β-Ionone	A45
1650cm-1	A23		

Supplementary Fig S1.

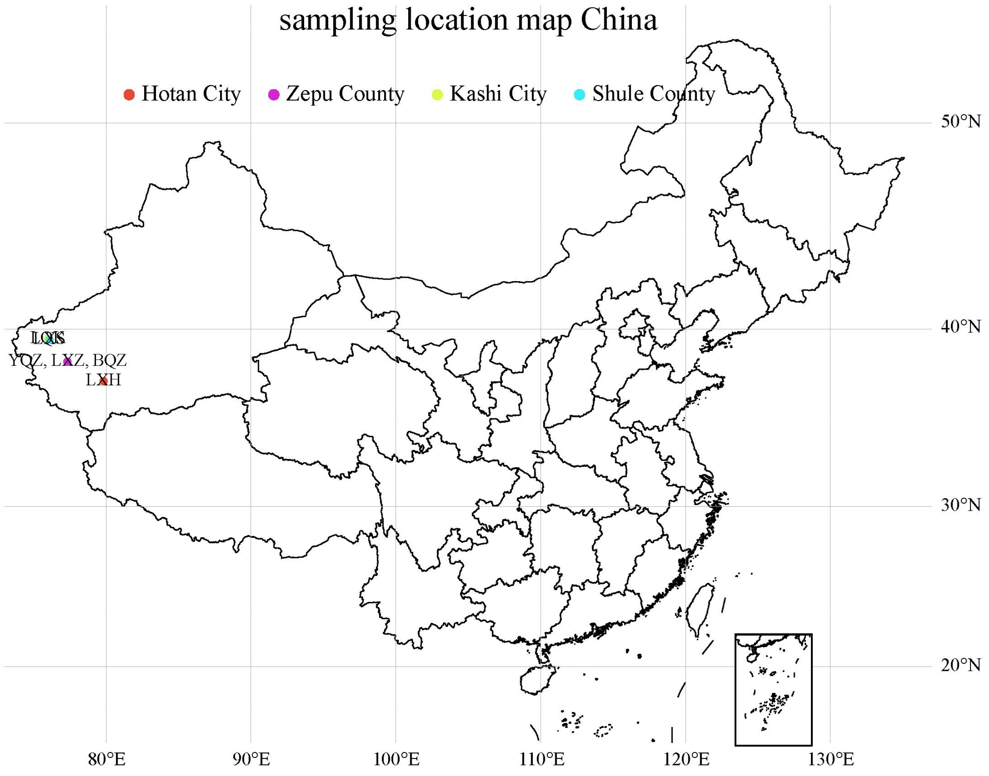

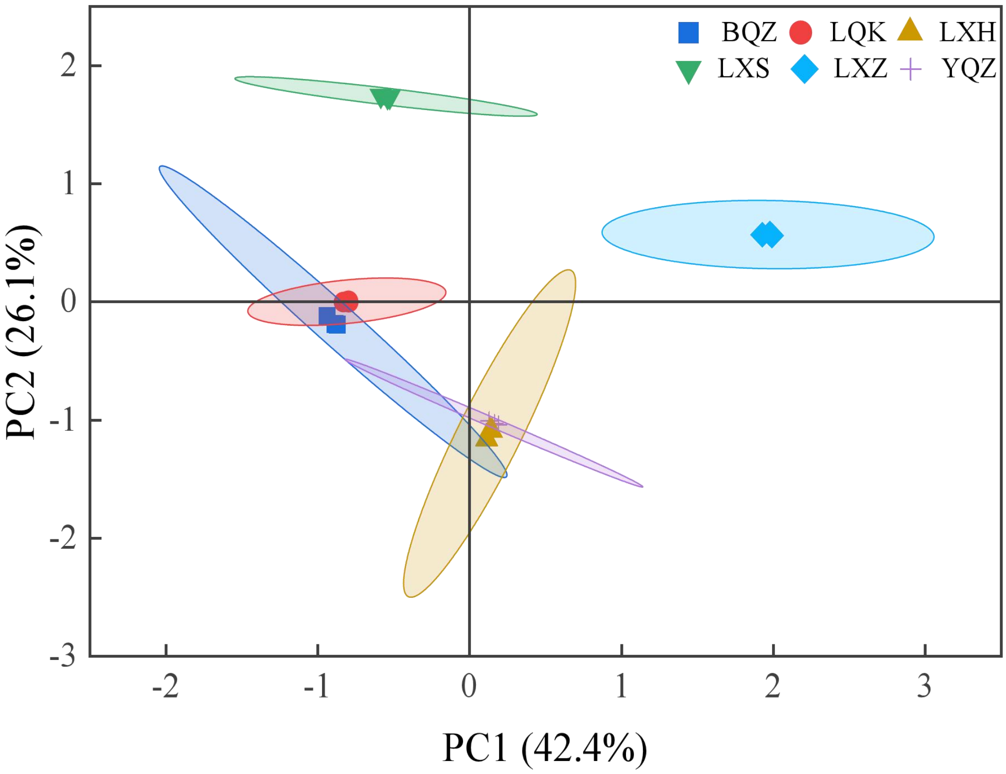

Supplementary Fig S2.

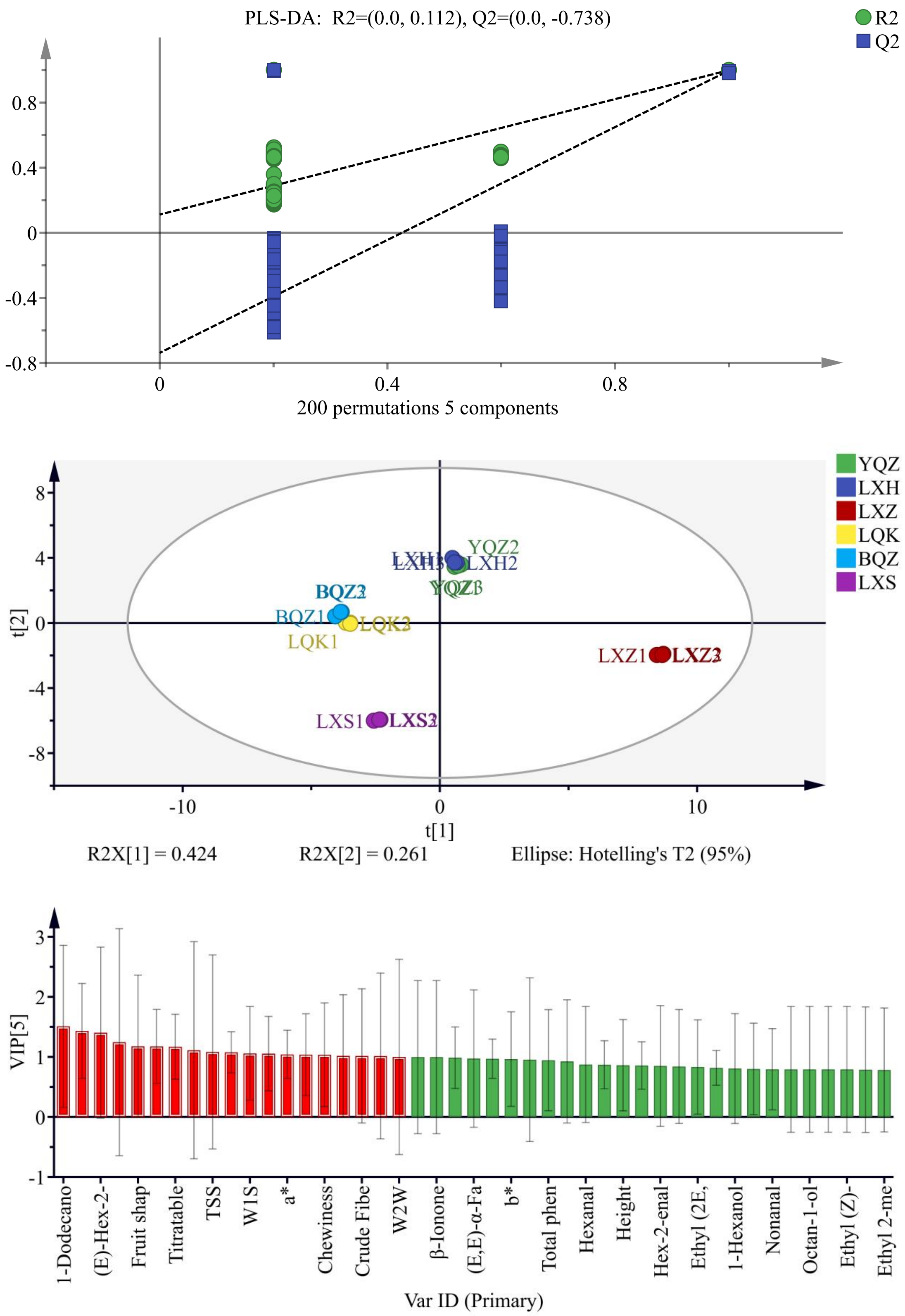

Supplement: Supplementary file 1 [file foods-15-02558-s001.zip › foods-4316966-supplementary.pdf]
